# Impact of implementation of the Dependency Act on the Spanish economy: an analysis after the 2008 financial crisis

**DOI:** 10.1007/s10754-021-09310-9

**Published:** 2021-08-04

**Authors:** Raúl Del Pozo-Rubio, Fernando Bermejo-Patón, Pablo Moya-Martínez

**Affiliations:** 1 Department of Economics and Finance, University of Castilla-La Mancha, Avda. Los Alfares, 44. 16.071, Cuenca, Spain; 2 Research Group Economy, Food and Society, University of Castilla-La Mancha, Cuenca, Spain

**Keywords:** Long-term care expenditure, Healthcare financing, Input–output model, Financial crisis, D57, H53, I38

## Abstract

The aim of this paper is to assess the industry-wide impact of Long-Term Care (LTC) spending on the Spanish economy. LTC spending includes beneficiaries’ copayment and the impact is quantified in terms of output, employment and value added. To this purpose, we use an input–output model of the Spanish economy that allows us to further describe how the value added generated is distributed throughout the economy according to the existing benefit-mix (in kind services, cash benefit for informal care and cash benefit for personal assistance). Additionally, the model provides results on how the return on LTC spending would improve by using only in-kind services instead of the benefit mix currently in place. The 2012 Spanish Input–Output Table at current prices was extracted from the WIOD Database’s 2016 Release. Consumption data for dependent, employed, and unemployed households were collected from the Spanish Household Budget Survey for 2012. The findings reveal that the total annual costs are 7,205.43 million €, with total costs from in-kind services being almost 71% higher than total costs from cash benefits. Each million euros invested in in-kind services and CBPA would create 41.91 jobs (68.41% direct, 9.16% indirect and 22.43% induced). However, each million euros spent on cash benefits would result in 16.88 jobs overall (53.02% direct, 24.53% indirect and 22.45% induced). The total number of jobs is 151,353 at the aggregate level, being 46,840 depending on cash-benefits and 104,513 on in-kind services.

**Supplementary Information:**

The online version contains supplementary material available at 10.1007/s10754-021-09310-9.

## Introduction

The process of population ageing is an inevitable challenge that European Union member states have to address, especially regarding the availability of public resources. The number of pensioners over the age of 65 is expected to increase in the EU from 18% in 2015 to 28% in 2060 and that of retirees over the age of 80 from 5 to 12% by 2060 (European Commission 2015). This implies a considerable growth in Long-Term Care (LTC), which can be defined as the set of needs that people require to carry out their day-to-day activities. (Colombo et al., 2011; Muir, 2017). In 2014, the average public spending on LTC in all OECD countries was 1.5% of GDP, ranging from more than 3% in Netherlands, Finland or Sweden and less than 1% in Spain or the United States (OECD, 2016).

To tackle this challenge in Spain, the Dependency Act was passed in 2006, with the main objective of catering for 1.26 million persons requiring LTC (Official Bulletin State, 2006). However, the financial recession that erupted after the collapse of Lehmann Brothers in 2008 was the starting point of difficult times for the world’s economy in general and for LTC systems in particular. The fiscal consolidation in Spain required difficult decisions regarding healthcare policies (Del Pino & Ramos, 2018). From 2012, in order to reduce the budget deficit and fulfil fiscal consolidation targets, strong structural fiscal measures were imposed that affected the Spanish LTC system (Ortega & Peñalosa, 2014; Official Bulletin State, 2012a, 2012b; Economic & Social Council of Spain, 2012; Economic & Social Council of Spain, 2013). This reform represented an austerity policy model, reducing public expenditure, decreasing intensity of services and delaying correct access to benefits. For example, LTC system benefits, amendments to unemployment benefits and the pensions reduced the total public spending ratio by 0.48% of GDP between 2011–2014 (Martí & Pérez, 2015).

The Spanish LTC system catalogue involves in-kind services (residential care, day/night care or home-help services) and cash benefits. The latter can be divided into cash benefit for personal assistance (CBPA) which could be used to either privately hire formal services (personal help, home care, or both help) or, most commonly, to subsidise informal carers, i.e., a cash benefit for informal care (CBIC). For each of these services, there is a copayment made by the households of the dependent persons and which represents an increase in the total expenditure on LTC (Del Pozo-Rubio et al., 2020). Therefore, it is important to understand that total LTC spending depends on the distribution (case-mix) of in-kind services, CBPA and CBIC financed through public spending and the copayment made by dependent households for each of the services (see Peña-Longobardo et al. (2016) for a thorough review of the Spanish LTC System).

To the best of our knowledge, most of the existing literature has mainly focused on using different econometric models to deal with financing or future spending projections (Moya-Martínez et al. 2020). None of the previous studies has estimated the economy-wide effect of LTC spending on the Spanish industry after the structural reform of 2012. Consequently, to fill this gap, the aim of this paper is to assess the economic impact of spending on LTC in Spain, considering its current configuration. This assessment is performed in terms of employment and income return on the consumption demand sustained by the LTC spending allocated for dependency. Additionally, the outcomes of this study provide insights into the distribution of the income generated, which allow us to ascertain how a different benefit-mix (in-kind services, CBPA and CBIC) could affect the country's economy structure. With this purpose, this study benefits from the main advantages of the IO framework that are described in (Miller & Blair, 2009). Broadly speaking, the main strength of IO models lies in two specific factors: firstly, its ability to quantify not only the direct impacts on production that are caused by an exogenous shock in demand (the LTC expenditure), but also the spillover effects on the local economy. Secondly, these models allow results to be obtained at a very detailed level of industry disaggregation. These effects are quantified in terms of the number of jobs required to sustain the production level and the value added generated by industry, which can be further split into the income distributed to different economic agents via wages, gross operating surplus, social contributions and net taxes on production. Besides these main advantages, when extending IO modelling to Demographic-Economic IO modelling, the effects of consumption by different population groups can be measured (here dependent persons, the employed and unemployed), which substantially improves the accuracy of the results (Batey, & Rose, 1990). All these advantages make IO models a suitable tool in different fields of research for measuring carbon footprints and energy intensities in environmental analysis, for the economic impact evaluation of tourism activities, as well as for sectoral analysis of policymaker decisions (see for instance (Ten Raa, 2017), papers collected in (Kurtz et al., 1998), and comments in (Carter, 2000)).

The structure of the paper is as follows. Section "Methodology and data" describes the main framework of the methodology and then the data used. In Sect. "Results", we present the results of the model output. The final section discusses the main policy implications of our findings and summarises the conclusions drawn.

## Methodology and data

### Main framework

To study the economy-wide effects of dependency costs (public and private spending) on the Spanish economy, we use input–output methodology. As previously stated, the advantage of the IO framework in modelling demand shocks lies in its ability to account for linkages between industries in general equilibrium, which allows spillover effects in the local economy to be determined. In doing so, IO models provide accurate results of the economic impact caused by such shocks on industry sectors at the highest level of disaggregation (64 branches according to NACE rev.2).^1^ Given an initial exogenous shock in demand (the direct impact on output), IO models further estimate indirect impacts (those arising from inter-industrial connections) and induced impacts (those arising from income-consumption connections).

Nonetheless, a significant limitation of basic IO models lies in their inability to deal with unemployment. As stated in Batey and Weeks (1989), by ignoring the consumption from the unemployed when output levels change, the induced effect of the demand shock can be underestimated. A way to mitigate such weakness is to extend the original IO framework in a class of models deemed demo-economic models (Batey, 2018), in which specific socio-demographic variables are included. Thus, these integrated models can explicitly consider local and in-migrant wages and consumption responses, as well as unemployment, social security benefits and contractual heterogeneity (Madden, 1993; Van Dijk & Oosterhaven, 1985).

All the above considered, the model applied in this study is built upon a first approach proposed in Bermejo and Del Pozo-Rubio (2019) and Moya-Martínez et al. (2020), which was extended here to explicitly consider consumption not only by employed households but also by unemployed households. Both sorts of households were then transferred to the inter-industry transaction matrix, behaving like any other industrial sector but in this case, their input was consumption and their output was labour.

**Fig. 1:**
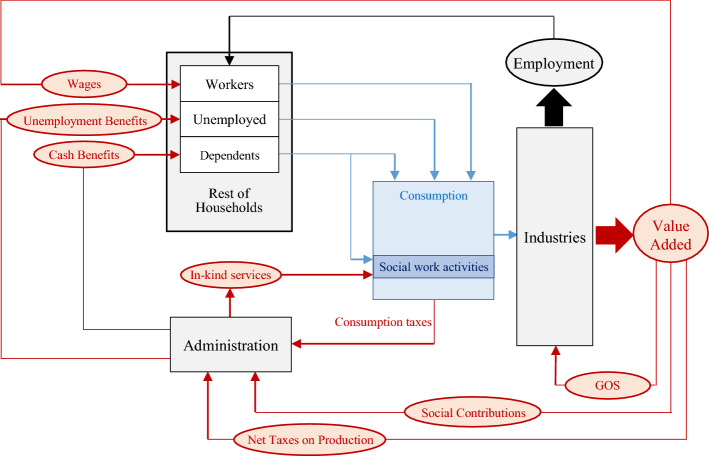
Modelling demo-economic impact of LTC spending. *Source*: Own preparation

As Fig. 1 shows, the allocation of public LTC and household co-payment on both cash benefits and in-kind services stimulates demand not only for social care activities but also for the rest of the economic sectors. This increase in consumption demand initially causes a proportional gain in gross output (direct impact) to be subsequently transmitted to the production system, where inter-industrial backward linkages generate a multiplicative effect (indirect impact). Besides industrial inputs, sectors also demand additional labour, increasing the total wage income in the economy consequently spent by those worker households, generating a subsequent round of consumption shocks (induced impact). Assuming a fixed labour force, increased demand for workers also decreases unemployment, leading to a rise in consumption due to the substitution effect between employed and unemployed households. Moreover, as Fig. 1 also illustrates, the value added generated from the production caused by the demand shock covers wages and social contributions, Gross Operating Surplus and other net taxes on production that constitute the income return on the dependency costs. Formally, in equilibrium, the IO model is defined as follows:

1 $$\left(\begin{array}{ccc}\mathbf{I}-\mathbf{A}& -{\mathbf{c}}_{\mathrm{E}}& -{\mathbf{c}}_{\mathrm{U}}{w}_{\mathrm{U}}\\ -{\mathbf{w}}_{\mathrm{x}}& 1& 0\\ {\mathbf{l}}_{\mathrm{d}}& 0& 1\end{array}\right)\times \left(\begin{array}{c}\mathbf{x}\\ {y}_{\mathrm{E}}\\ u\end{array}\right)=\left(\begin{array}{c}{\mathbf{c}}^{\mathrm{D}}+{\mathbf{f}}^{\mathrm{O}}\\ {y}_{\mathrm{S}}\\ p\end{array}\right)$$

where: $\mathbf{A}$ is the direct input requirement matrix from Spain. ${\mathbf{c}}_{\mathrm{E}}$ is a (column) vector of consumption coefficients for employed persons. ${\mathbf{c}}_{\mathrm{U}}$ is a (column) vector of consumption coefficients for unemployed persons. ${\mathbf{c}}^{\mathrm{D}}$ is a (column) vector of monetary consumption from dependency costs. ${\mathbf{w}}_{\mathrm{x}}$ is a (row) vector of annual wages by sectoral domestic output. ${w}_{\mathrm{U}}$ is the average annual welfare benefit of an unemployed person. ${\mathbf{l}}_{\mathrm{d}}$ is a (row) vector of labour demand coefficients (jobs/million euros). $\mathbf{x}$ is a (column) vector of the total domestic output by sector. ${\mathbf{f}}^{\mathrm{O}}$ is a (column) vector of final demand, excluding consumption of employed, unemployed and dependent persons. ${y}_{\mathrm{E}}$ is the total income for employed persons. ${y}_{\mathrm{S}}$ is the total income from exogenous sources to households of employed persons. $u$ is the total number of unemployed persons. $p$ is the total labour force.

The model can be expressed as a system of equations given by:

2 $$\left(\mathbf{I}-\mathbf{A}\right)\mathbf{x}-{\mathbf{c}}_{\mathrm{E}}{y}_{\mathrm{E}}-{\mathbf{c}}_{\mathrm{U}}{w}_{\mathrm{U}}u={\mathbf{c}}^{\mathrm{D}}+{\mathbf{f}}^{\mathrm{O}}$$

3 $$-{\mathbf{w}}_{\mathrm{x}}\mathbf{x}+{y}_{\mathrm{E}}={y}_{\mathrm{S}}$$

4 $${\mathbf{l}}_{\mathrm{d}}\mathbf{x}+u=p$$

Equation 2 represents the supply–demand balance in the economy, in which total production minus intermediate, employed and unemployed consumption is equal to final demand. For the purpose of this investigation, final demand is split into the consumption funded with dependency costs and the remaining final demand. Equation 3 implies that total income is total wage income plus any external income for employed households. Equation 4 guarantees the demographic balance, in which total employment plus total unemployment is equal to the available labour force. As noted in Batey & Madden (1999), the latter reflects the reduction (or increase) in unemployed workers resulting from an increase (or decrease) in industrial production. The equilibrium solution for total output is given by:

5 $$\mathbf{x}={\left(\mathbf{I}-\mathbf{A}-{\mathbf{c}}_{\mathrm{E}}{\mathbf{w}}_{\mathrm{x}}+{\mathbf{c}}_{\mathrm{U}}{w}_{\mathrm{U}}{\mathbf{l}}_{\mathrm{d}}\right)}^{-1}\times \left({\mathbf{c}}^{\mathrm{D}}+{\mathbf{f}}^{\mathrm{O}}+{\mathbf{c}}_{\mathrm{U}}{w}_{\mathrm{U}}p\right)$$

In order to estimate the effect that the implementation of dependency costs may cause in a given year (${\mathbf{x}}^{\mathrm{D}}$), we shock the economy with the full final demand (${\mathbf{f}}^{\mathrm{O}}$) minus the exogenous consumption (${\mathbf{c}}^{\mathrm{D}}$), and subtract these results (${\mathbf{x}}^{\mathrm{O}}$) from the baseline ($\mathbf{x}$) obtained just with the full demand:

6 $${\mathbf{x}}^{\mathrm{D}}=\mathbf{x}-{\mathbf{x}}^{\mathrm{O}}={\left(\mathbf{I}-\mathbf{A}-{\mathbf{c}}_{\mathrm{E}}{\mathbf{w}}_{\mathrm{x}}+{\mathbf{c}}_{\mathrm{U}}{w}_{\mathrm{U}}{\mathbf{l}}_{\mathrm{d}}\right)}^{-1}\times {\mathbf{c}}^{\mathrm{D}}$$

where ${(\mathbf{I}-\mathbf{A}-{\mathbf{c}}_{\mathrm{E}}{\mathbf{w}}_{\mathrm{x}}+{\mathbf{c}}_{\mathrm{U}}{w}_{\mathrm{U}}{\mathbf{l}}_{\mathrm{d}})}^{-1}$ is the extended inverse matrix to capture the total effects (direct, indirect and induced) on output ${\mathbf{x}}^{\mathrm{D}}$ as a result of a particular change in the exogenous consumption (column) vector ${\mathbf{c}}^{\mathrm{D}}$. Such total effects are based on the inter-industry structure given by the technical coefficients in matrix $\mathbf{A}$ and the induced consumption by both the employed households $\left({\mathbf{c}}_{\mathrm{E}}{\mathbf{w}}_{\mathrm{x}}\right)$ and the unemployed households $\left({\mathbf{c}}_{\mathrm{U}}{w}_{\mathrm{U}}{\mathbf{l}}_{\mathrm{d}}\right)$.

In describing changes due to exogenous consumption, it is worth noting that monetary value ${\mathbf{c}}^{\mathrm{D}}$ is directly influenced by the type of assistance considered: in-kind services or cash benefits (CBPA and CBIC). Both the Administration^2^ and the dependent households (co-payment) pay directly for the social work assistance demanded when in-kind services are provided. In this particular case, the co-payment of households results in a reduction of the income level allocated to dependent persons’ consumption. Accordingly, column (vector) ${\mathbf{c}}^{\mathrm{D}}$ will include the in-kind services expenditure in the sector of social work activities minus a negative impact on the overall dependent persons’ consumption basket due to co-payment.

When cash benefits are implemented, the Administration provides the eligible households with an amount of money to meet the dependency needs. Furthermore, as mentioned in the introduction, two different sorts of economic benefits must be considered: CBPA and CBIC, accounting respectively for 13.36 and 86.64% of total cash benefits (Del Pozo-Rubio & Jiménez-Rubio, 2019). Firstly, CBPA entails the obligation for dependent households to allocate both the benefits received and co-payment to formal care. This actually implies a similar outcome to the above-mentioned for in-kind services, which means that column (vector) ${\mathbf{c}}^{\mathrm{D}}$ will contain the CBPA benefit directly assigned to the sector of social work activities minus an overall drop in dependents’ consumption due to co-payment. On the other hand, CBIC implementation involves those payments from the Spanish LTC System as an economic acknowledgement of informal care. While these sorts of transfers are provided to meet the dependency needs, the ultimate effect is an increase in the total income of dependent households. Assuming that informal care is issued within the family circle (Del Pozo-Rubio et al., 2017), no third-party payment is made for this service, and then this rise in income share will effectively ease the budget constraint of households, allowing them to spend a larger amount of money not only on social work activities but also on the rest of goods and services.

Once ${\mathbf{x}}^{\mathrm{D}}$ is known, both total (direct, indirect, and induced) employment (${\mathbf{l}}^{\mathrm{D}}$) and value added (${\mathbf{v}}^{\mathrm{D}}$) sustained by dependent persons can be obtained as follows:

7 $${\mathbf{l}}^{\mathrm{D}}={\widehat{\mathbf{l}}}_{\mathrm{d}}\times {\mathbf{x}}^{\mathrm{D}}={\widehat{\mathbf{l}}}_{\mathrm{d}}\times {\left(\mathbf{I}-\mathbf{A}-{\mathbf{c}}_{\mathrm{E}}{\mathbf{w}}_{\mathrm{x}}+{\mathbf{c}}_{\mathrm{U}}{w}_{\mathrm{U}}{\mathbf{l}}_{\mathrm{d}}\right)}^{-1}\times {\mathbf{c}}^{\mathrm{D}}$$

8 $${\mathbf{v}}^{\mathrm{D}}={\widehat{\mathbf{v}}}_{\mathrm{d}}\times {\mathbf{x}}^{\mathrm{D}}={\widehat{\mathbf{v}}}_{\mathrm{d}}\times {\left(\mathbf{I}-\mathbf{A}-{\mathbf{c}}_{\mathrm{E}}{\mathbf{w}}_{\mathrm{x}}+{\mathbf{c}}_{\mathrm{U}}{w}_{\mathrm{U}}{\mathbf{l}}_{\mathrm{d}}\right)}^{-1}\times {\mathbf{c}}^{\mathrm{D}}$$

where ${\widehat{\mathbf{l}}}_{\mathrm{d}}$ and ${\widehat{\mathbf{v}}}_{\mathrm{d}}$ are diagonal matrices obtained from vectors ${\mathbf{l}}_{\mathbf{d}}=\frac{{\mathbf{l}}_{j}}{{\mathbf{X}}_{j}}$ and ${\mathbf{v}}_{\mathbf{d}}=\frac{{\mathbf{v}}_{j}}{{\mathbf{X}}_{j}}$, that represent the direct coefficients of sectoral employment $\mathbf{l}$ and sectoral value added $\mathbf{v}$ respectively.

### Data

The input data for the IO model are public spending and contributions or co-payments of dependent households on LTC, split into cash benefits and in-kind services. These data and their methodology have recently been detailed elsewhere (Del Pozo-Rubio & Jiménez-Rubio, 2019).

The 2012 Symmetric IO Table at current prices for Spain was extracted from the WIOD Database’s 2016 Release (Timmer et al., 2012, 2015). The last release of this database covers 28 EU countries plus 15 other major countries in the world for the period from 2000 to 2014. The Symmetric IO Table is defined at producer prices and adheres to the 2008 version of the System of National Accounts. The information is disaggregated in 56 sectors according to the International Standard Industrial Classification, revision 4. Following the appropriate methodology, we extracted the Symmetric IO Table for the Spanish economy in 2012 to further aggregate data in 16 sectors.

Consumption data for dependent, employed, and unemployed households were collected from the Spanish Household Budget Survey for 2012 (National Statistics Institute of Spain, 2012a). This database provides information on the amount (in purchasing prices) and structure of household expenditures according to Classification of Individual Consumption by Purpose. The Household Budget Survey also contains socioeconomic data about the standard of living, income and the professional activity of the household reference person. For simplicity, we shall assume that a household is classified according to the status of its reference person. These values were transformed into producer prices by removing trade and transportation margins, and indirect taxes to products (including value added taxes among others).

Given that Household Budget Survey microdata refer to commodity expenditures, it was necessary to allocate these commodities to the industrial sectoring scheme adopted by the Symmetric IO Table to provide comparable results between consumption purposes and sectoral production. It is important to note that there is no official bridge between the two classification systems used in the Household Budget Survey and the Symmetric IO Table (i.e., Classification of Individual Consumption by Purpose and NACE rev.2). Therefore, in order to distribute the consumption shares, both the Symmetric IO Table and Household Budget Survey were aggregated into 16 sectors according to the International Standard Industrial Classification (rev. 4) aggregation (see Table S1 for Symmetric IO Table and Table S2 for Household Budget Survey in the online supplementary material). For the purpose of this investigation, health services and social work activities (sector Q in International Standard Industrial Classification rev. 4) were split into two major divisions of Health services and Social work activities, sector S14 and sector S15, respectively, in the classification for this paper.

A last important feature involves the imports incorporated into intermediate input flows and final demand. While imports of intermediate inputs are necessary for domestic production, imported products cause no effective impact on the generation of both domestic value added and employment. Therefore, imports into final demand consumption were excluded in this paper. Moreover, considering that the Household Budget Survey does not provide information about expenditures related to imports, the distinction between domestic and imported products for household consumption is based upon the corresponding distribution of domestic and imported products for the household consumption in the Symmetric IO Table.

Finally, sectoral value added and all its components (wages, social contributions, gross operating surplus and net taxes on production), as well as the sectoral number of workers, were extracted from the National Accounting of the Spanish Statistics Institute (National Statistics Institute of Spain, 2012b).

## Results

First, we consider the expenditure on LTC that has funded the demand shock triggering the economic return to be analysed. Total annual LTC costs were €7,205.43 million in 2012, with total costs from in-kind services (€4,545.85 million) being almost 71% larger than total costs from cash benefits (CBPA and CBIC). Furthermore, CBPA only represents 4.93% of total LTC spending. Regarding the share of spending between the administration (public spending) and the households of dependents (co-payment), we can observe that all benefits are equally distributed, ranging between 50.9 and 54.8% of the expenditure for the public administration (See Table S3 in the online supplementary material).

In a second stage, the household consumption derived from the above costs of dependency was estimated (Table S4 in the online supplementary material). We used the Household Budget Survey microdata for 2012, containing total statistics of the household consumption expenditure over a sample of roughly 24,000 records. By defining specific data filters based on the main source of household income, we were able to determine the consumption profile for dependent households (column ${\mathbf{c}}_{\mathrm{D}}$ in Table S4). The monetary value of the exogenous consumption (columns ${\mathbf{c}}^{\mathbf{D}}$ in Table S4) was then obtained by applying coefficients ${\mathbf{c}}_{\mathrm{D}}$ to the total costs that both the administration and dependent households allocate for consumption according to each type of benefit. Columns ${\mathbf{c}}^{\mathbf{D}}$ are shown in producer prices and exclude imports, which explains that the aggregated values are €189.62 million for CBPA (53.4% of the initial costs of €355.42 million), €2,077.26 million for CBIC (90.1% of the initial costs of €2,305.12 million) and €2,584.10 million for in-kind services (56.9% of the initial costs of €4,544.85 million). The consumption of employed and unemployed households was obtained by following a similar procedure with the Household Budget Survey microdata. In this case, we identify the consumption profiles of both types of households, which are shown in columns ${\mathbf{c}}_{\mathrm{E}}$ (employed) and ${\mathbf{c}}_{\mathrm{U}}$ (unemployed) in Table S4.

As Table S4 shows, the consumption profile of dependent households (${\mathbf{c}}_{\mathrm{D}}$) is skewed toward necessity goods (S6-Wholesale and retail trade, S8-Accommodation and food services, and S11-Real estate services), which account for more than 50% of the total expenditure for dependent persons. Employed households show a higher level of consumption in S8-Accommodation and food service activities and lower expenditure in S11-Real estate activities, while the unemployed show the highest level of consumption for S11-Real estate activities with lower expenditures in S8-Accommodation and food service activities. It is important to note the similarity between employed and unemployed consumption shares, reflecting a certain continuity in consumption preferences, so the substitution effect for the unemployed mainly relies on a significantly higher expenditure on the real estate sector and lower on accommodation and food service activities as well as on arts, entertainment and recreation.

Table 1 shows both total output ${\mathbf{x}}^{\mathrm{D}}$ and value added ${\mathbf{v}}^{\mathrm{D}}$ generated from the consumption described above. The results are presented according to the type of LTC benefits considered. First, output ${\mathbf{x}}^{\mathrm{D}}$ is estimated by using Eq. (6), being €10,266.88 million in total when all LTC benefits are aggregated. More specifically, cash benefits account for 4.13% and 39.97% of CBPA and CBIC respectively, while 55.9% are related to in-kind services. Once the output is obtained, the valued added ${\mathbf{v}}^{\mathrm{D}}$ estimated by using Eq. (8) corresponds to €5,900.09 million, which entails the total income generated from the previous expenditure on consumption to be further distributed among wages, gross operating surplus and a fiscal return via social contributions and net taxes on production. The total value added comes from CBPA in 4.22%, CBIC in 38.81% and in-kind services in 59.96%.

**Table 1:** Total output ${\mathrm{x}}^{\mathrm{D}}$ and value added ${\mathrm{v}}^{\mathrm{D}}$ returned from the expenditure on consumption funded by LTC spending (2012)

Sector	CBPA		CBIC		In-kind services		Global	
	Total output ${\mathbf{x}}^{\mathrm{D}}$	Total value added ${\mathbf{v}}^{\mathrm{D}}$	Total output ${\mathbf{x}}^{\mathrm{D}}$	Total value added ${\mathbf{v}}^{\mathrm{D}}$	Total output ${\mathbf{x}}^{\mathrm{D}}$	Total Value added ${\mathbf{v}}^{\mathrm{D}}$	Total output ${\mathbf{x}}^{\mathrm{D}}$	Total value added ${\mathbf{v}}^{\mathrm{D}}$
S1	1.41	0.74	73.92	38.90	23.66	12.45	98.99	52.09
S2	5.42	1.86	259.07	89.11	89.22	30.69	353.71	121.66
S3	8.05	1.76	288.53	62.98	125.11	27.31	421.68	92.04
S4	27.33	6.66	301.24	73.40	372.55	90.78	701.12	170.84
S5	2.72	1.20	97.54	43.07	42.27	18.67	142.54	62.94
S6	8.22	4.79	659.58	383.80	155.83	90.68	823.63	479.27
S7	5.60	2.26	201.93	81.56	87.17	35.21	294.70	119.03
S8	5.56	3.21	444.68	256.76	105.26	60.78	555.50	320.75
S9	4.43	2.15	162.72	78.97	69.18	33.57	236.33	114.69
S10	3.18	1.83	215.01	123.96	57.21	32.98	275.40	158.77
S11	− 24.96	− 21.43	732.72	629.08	− 262.80	− 225.63	444.96	382.03
S12	12.56	7.13	200.06	113.49	176.00	99.84	388.62	220.46
S13	3.52	2.75	106.68	83.40	53.17	41.57	163.37	127.72
S14	12.48	7.98	99.66	63.68	167.27	106.87	279.42	178.53
S15	348.36	226.00	52.90	34.32	4458.22	2892.28	4859.47	3152.60
S16	0.32	0.21	207.11	133.59	20.01	12.91	227.44	146.70
Total	424.21	249.10	4103.35	2290.05	5739.31	3360.95	10266.88	5900.09

*Source*: Authors’ own calculations from Household Budget Survey data [51]

Values are denoted in million euros

S1: Agriculture, forestry and fishing; S2: Energy, water and waste collection; S3: Food and textile; S4: Manufacturing; S5: Construction; S6: Wholesale and retail trade; S7: Transport; S8: Accommodation and food service activities; S9: Information and communication; S10: Financial and insurance activities; S11: Real estate activities; S12: Professional, scientific and technical activities; S13: Public administration, defence and education; S14: Health services; S15: Social work activities; S16: Arts, entertainment, recreation and other services

*CBPA* Cash benefit for personal assistance, *CBIC* Cash benefit for informal care

${\mathbf{x}}^{\mathbf{D}}$, ${\mathbf{v}}^{\mathbf{D}}$: Million euros of production and value added in producer prices according to the different types of LTC benefits

Table 2 shows total employment ${\mathbf{l}}^{\mathrm{D}}$ (direct, indirect and induced) required to obtain total output ${\mathbf{x}}^{\mathrm{D}}$. The total number of jobs is 151,353, with 46,840 depending on cash-benefits (7,940 on CBPA and 38,900 on CBIC), and 104,513 on in-kind services, 69.05% of the total employment. Direct and indirect employment account, respectively, for 69% and 9% of total employment when CBPA is applied. The share of direct and indirect employment remains unchanged when in-kind services are applied. Moreover, induced employment is roughly 22% of total employment for the three types of benefits considered. Interestingly enough, direct employment falls to 53% of total employment and indirect employment rises to 24.5% when CBIC is implemented, more than double the amount generated by the rest of the benefits.

**Table 2:** Employment (direct, indirect, induced and total) depending on the consumption funded with LTC spending (2012)

Sector	CBPA				CBIC				In-kind services				Global
	l_dir_	l_indir_	l_indu_	${\mathbf{l}}^{\mathrm{D}}$	l_dir_	l_indir_	l_indu_	${\mathbf{l}}^{\mathrm{D}}$	l_dir_	l_indir_	l_indu_	${\mathbf{l}}^{\mathrm{D}}$	${\mathbf{l}}^{\mathrm{D}}$
S1	− 21	− 15	58	23	271	638	285	1195	− 242	− 141	765	382	1600
S2	− 15	5	21	11	193	249	102	544	− 172	84	275	187	743
S3	− 23	− 3	57	31	306	516	279	1100	− 272	1	748	477	1608
S4	− 19	63	62	106	246	623	303	1173	− 219	856	814	1450	2729
S5	− 17	9	29	22	218	416	144	779	− 194	144	388	338	1139
S6	− 548	209	479	140	7252	1592	2346	11189	− 6453	2797	6300	2644	13972
S7	− 28	− 11	84	44	375	811	409	1595	− 334	− 77	1099	689	2328
S8	− 268	35	297	65	3543	180	1458	5180	− 3152	463	3916	1226	6472
S9	− 26	13	37	24	347	337	180	864	− 309	193	484	367	1255
S10	− 27	− 5	49	18	362	580	242	1184	− 322	− 14	651	315	1517
S11	− 60	− 2	27	− 36	800	114	133	1047	− 712	− 22	358	− 376	636
S12	− 35	99	148	212	467	2178	724	3370	− 416	1435	1946	2965	6546
S13	− 70	19	113	62	920	411	552	1882	− 818	274	1482	938	2883
S14	− 63	167	47	150	838	130	228	1197	− 746	2141	614	2009	3355
S15	6960	77	25	7061	891	60	121	1072	89055	987	326	90368	98501
S16	− 272	31	249	9	3593	714	1220	5527	− 3197	455	3277	534	6070
Total	5468 (68.9%)	691 (8.7%)	1781 (22.4%)	7940	20621 (53.0%)	9550 (24.5%)	8728 (22.4%)	38900	71498 (68.4%)	9575 (9.2%)	23441 (22.4%)	104513	151353

*Source*: Authors’ own calculations from Household Budget Survey data [51]

S1: Agriculture, forestry and fishing; S2: Energy, water and waste collection; S3: Food and textile; S4: Manufacturing; S5: Construction; S6: Wholesale and retail trade; S7: Transport; S8: Accommodation and food service activities; S9: Information and communication; S10: Financial and insurance activities; S11: Real estate activities; S12: Professional, scientific and technical activities; S13: Public administration, defence and education; S14: Health services; S15: Social work activities; S16: Arts, entertainment, recreation and other services

*CBPA* Cash benefit for personal assistance, *CBIC* Cash benefit for informal care

${{\mathbf{l}}^{\mathrm{D}}}_{\mathbf{d}\mathbf{i}\mathbf{r}}$: Direct employment; ${{\mathbf{l}}^{\mathrm{D}}}_{\mathbf{i}\mathbf{n}\mathbf{d}\mathbf{i}\mathbf{r}}$: Indirect employment; ${{\mathbf{l}}^{\mathrm{D}}}_{\mathbf{i}\mathbf{n}\mathbf{d}\mathbf{u}}$: Induced employment; ${\mathbf{l}}^{\mathrm{D}}$: Total employment

The last column in Table 2 shows the combined effect of all LTC benefits on employment. As expected, S15-social work activities exhibit the highest level of employment, which is consistent with the direct impact on output caused by LTC spending. Nevertheless, a significant increase is also observed in services, such as S6-Wholesale and retail trade, S8-Accommodation and food, S12-Professional, administrative and support activities and S16- Arts, entertainment and recreation services. This is mainly explained by the indirect and induced effects of LTC spending, that is the increase in production of those sectors providing inputs to social work activities and those sectors providing goods and services for the consumption of the employed depending on LTC spending.

Table 3 shows the distribution of value added ${\mathbf{v}}^{\mathrm{D}}$ according to LTC benefits. In light of these results, it is important to mention that CBPA exhibits a similar income distribution to that of in-kind services, with both of them being significantly skewed towards the remuneration of labour (roughly 81% of value added) at the expense of gross operating surplus (22%). This can be explained by two combined effects: firstly, the effect of allocating benefits to a considerably labour-intensive sector like social work activities, and secondly, the negative impact of household co-payment on the basket consumption by dependents. Such a distribution clearly differs from the income shares when CBIC are applied, where gross operating surplus accounts for 56% of total value added while wages plus social contributions just add up to 42%.

**Table 3:** Distribution of income return on the consumption demand funded with LTC spending (2012)

Sector	Cash benefit for personal assistance (CBPA)				Cash benefit for informal care (CBIC)				In-kind services			
	Wages	Social contrib	GOS	NToP	Wages	Social contrib	GOS	NToP	Wages	Social contrib	GOS	NToP
S1	0.12	0.01	0.77	− 0.17	6.19	0.79	40.69	− 8.77	1.98	0.25	13.03	− 2.81
S2	0.42	0.10	1.31	0.03	20.12	4.99	62.46	1.54	6.93	1.72	21.51	0.53
S3	0.70	0.16	0.90	0.00	25.04	5.82	32.15	− 0.03	10.86	2.52	13.94	− 0.01
S4	3.20	0.83	2.65	− 0.02	35.25	9.12	29.21	− 0.18	43.60	11.27	36.13	− 0.22
S5	0.47	0.13	0.58	0.02	16.92	4.52	20.78	0.85	7.33	1.96	9.00	0.37
S6	2.27	0.61	1.90	0.01	181.82	48.67	152.41	0.91	42.96	11.50	36.01	0.21
S7	1.00	0.27	0.99	0.00	36.07	9.76	35.69	0.03	15.57	4.21	15.41	0.01
S8	1.29	0.20	1.71	0.01	103.13	15.63	137.08	0.92	24.41	3.70	32.45	0.22
S9	0.85	0.22	1.07	0.02	31.11	7.94	39.18	0.75	13.22	3.37	16.66	0.32
S10	0.73	0.25	0.70	0.16	49.21	16.80	47.47	10.47	13.09	4.47	12.63	2.79
S11	− 0.66	− 0.17	− 19.03	− 1.58	19.30	4.94	558.60	46.24	− 6.92	− 1.77	− 200.35	− 16.59
S12	3.82	1.05	2.29	− 0.03	60.77	16.73	36.44	− 0.45	53.46	14.72	32.06	− 0.40
S13	1.61	0.50	0.64	0.00	48.87	15.03	19.37	0.13	24.36	7.49	9.66	0.06
S14	4.91	1.24	1.81	0.01	39.24	9.93	14.43	0.08	65.85	16.66	24.22	0.13
S15	136.54	39.08	53.30	− 2.92	20.73	5.93	8.09	− 0.44	1,747.44	500.16	682.10	− 37.41
S16	0.12	0.02	0.07	0.00	75.09	14.98	43.45	0.06	7.26	1.45	4.20	0.01
Total	157.38 (63.2%)	44.50 (17.9%)	51.66 (20.7%)	− 4.44 (− 1.8%)	768.85 (33.6%)	191.57 (8.4%)	1,277.51 (55.8%)	52.11 (2.3%)	2,071.40 (61.6%)	583.69 (17.4%)	758.64 (22.6%)	− 52.78 (− 1.6%)

*Source:* Authors’ own calculations from Household Budget Survey data [51]

Values are denoted in million euros

S1: Agriculture, forestry and fishing; S2: Energy, water and waste collection; S3: Food and textile; S4: Manufacturing; S5: Construction; S6: Wholesale and retail trade; S7: Transport; S8: Accommodation and food service activities; S9: Information and communication; S10: Financial and insurance activities; S11: Real estate activities; S12: Professional, scientific and technical activities; S13: Public administration, defence and education; S14: Health services; S15: Social work activities; S16: Arts, entertainment, recreation and other services

*GOS* Gross operating surplus, *NToP* Net taxes on production

As a summary of the outcomes shown in the previous tables, Figure S1 (online supplementary material) reports the level of output, income and employment generated from every million euros of LTC expenditure depending on the type of benefits considered (CBPA, CBIC and in-kind services). The results prove the multiplier effect triggered by the initial spending on LTC captured by the IO model. In particular, such results evidence a somewhat similar return from CBPA and in-kind services (slightly better in all variables for CBPA except gross operating surplus). Thus, every million euros allocated to CBPA returned 2.34 million euros in output, sustained 43.83 jobs and generated 1.37 million euros in value added on production to be split into wages (0.87 million euros), gross operating surplus (0.29 million euros), social contributions (0.25 million euros) and net taxes on production (−0.02 million euros). Regarding in-kind services, every million euros in LTC returned 2.30 million euros in output, 41.91 jobs, while the value added on production was 1.35 split into wages (0.83 million euros), gross operating surplus (0.30 million euros), social contributions (0.23 million euros) and net taxes on production (−0.02 million euros). Nonetheless, one million euros allocated to CBIC brings a lower return in terms of output (1.78 million euros), 16.88 jobs, and value added on production (0.99 million euros) split into wages (0.33 million euros), gross operating surplus (0.55 million euros), social contributions (0.08 million euros) and net taxes on production (0.02 million euros). Two apsects need to be considered when CBIC are applied: the employment required is reduced by almost 60% and the distribution of value added is significantly skewed towards gross operating surplus.

In light of the above, the distribution of value added according to the nature of the LTC benefits seems to be particularly remarkable. In this sense, CBPA and in-kind services give priority to wages (63.2% and 61.6%, respectively) and social contributions (17.9% and 17.4%, respectively), with a less specific weight of gross operating surplus (20.7% for CBPA and 22.6% for in-kind services), whilst CBIC gives preference to gross operating surplus (55.8%) at the expense of both wages (33.6%) and social contributions (8.4%).

Before the analysis performed with our 2012 data, the Act by which the Spanish LTC System was created conferred a clear preference in providing in-kind services vs cash benefits. Due to the greater ease of implementation of the cash-benefits, this was not the case (Cervera et al., 2009; Del Pozo Rubio & Escribano Sotos, 2012). In the current situation, our data shows that in-kind services account for 63.08% of total LTC spending. Hence, a simulation analysis based on the type of LTC benefit granted becomes extremely important in order to establish the potential economic consequences arising from a change in the current structure of the LTC System. Table 4 compares the economic return of LTC spending when the mix of CBPA, CBIC and in-kind services is granted with that resulting from LTC being exclusively provided by in-kind services. The last column in Table 4 shows the variation both in absolute terms and in percentages. First, the total output generated by LTC spending would have increased from 10.27 to 14.69 million euros, giving rise to an increase in value added from 5.90 to 8.60 million euros as well as in the number of jobs from 151,353 to 267,899. Besides this quantitative gain in economic returns, the allocation of LTC spending exclusively on in-kind services would result in a distribution of value added rather different than that obtained from the current mix of LTC benefits. This means the economic return on LTC spending would be more skewed toward labour, so wages would account for 61.7% instead of 50.8% of the value added generated and the social contributions arising from such wages would also increase from 13.9% to 17.4% of the value added. This shift toward labour would occur at the expense of the GOS, which would decrease from 35.4% to 22.5% of the value added obtained. The results in Table 4 also evidence a slight shift from indirect to direct jobs when exclusively in-kind services are granted. Nevertheless, beyond this trade-off by 4% between both types of benefits as regards indirect or direct jobs, the simulation herein undertaken concludes that in-kind services would have been more effective in terms of employment and income generation than cash benefits.

**Table 4:** Simulation analysis of the demo-economic return on LTC spending (2012)

	Mix CBPA ⊕ CBIC ⊕ In-kind services		Exclusively In-kind services		Δ
Total output ${\mathrm{x}}^{\mathrm{D}}$		10266.88		14688.01	4421.13
Total Value added ${\mathrm{v}}^{\mathrm{D}}$		5900.09		8602.66	2702.57
Wages	50.8%	2997.63	61.7%	5309.64	2.312.01
Social Contributions	13.9%	819.76	17.4%	1496.48	676.72
GOS	35.4%	2087.81	22.5%	1932.70	− 155.11
Net Taxes on Production	− 0.1%	− 5.11	− 1.6%	− 136.16	− 131.05
Total number of jobs ${{\mathrm{l}}^{\mathrm{D}}}_{\mathrm{tot}}$		151353		267899	116546
${{\mathbf{l}}^{\mathrm{D}}}_{\mathbf{d}\mathbf{i}\mathbf{r}}$	64.5%	97587	68.4%	183341	85.754
${{\mathbf{l}}^{\mathrm{D}}}_{\mathbf{i}\mathbf{n}\mathbf{d}\mathbf{i}\mathbf{r}}$	13.1%	19816	9.1%	24472	4.656
${{\mathbf{l}}^{\mathrm{D}}}_{\mathbf{i}\mathbf{n}\mathbf{d}\mathbf{u}}$	22.4%	33950	22.4%	60086	26.136

Monetary values are denoted in million euros

*GOS* Gross operating surplus, *NToP* Net taxes on production

${{\mathbf{l}}^{\mathrm{D}}}_{\mathbf{d}\mathbf{i}\mathbf{r}}$: Direct employment; ${{\mathbf{l}}^{\mathrm{D}}}_{\mathbf{i}\mathbf{n}\mathbf{d}\mathbf{i}\mathbf{r}}$: Indirect employment; ${{\mathbf{l}}^{\mathrm{D}}}_{\mathbf{i}\mathbf{n}\mathbf{d}\mathbf{u}}$: Induced employment; ${\mathbf{l}}^{\mathrm{D}}$: Total employment

Finally, the outcomes of this simulation are supplemented by Table S5 (online supplementary material), where output, income (as value added split into its components) and employment (direct, indirect and induced) are provided at a sectoral level considering exclusively in-kind services had been granted. When comparing Table S5 with Tables 1 and 2, it is clear that the drop in output, income and employment due to applying the mix of CBPA, CBIC and in-kind services instead of applying exclusively in-kind services is mainly based on a significant underdevelopment of S15-social work activities. This decrease in S-15 is found together with lower values in S-4 Manufacturing, S-12 Professional, scientific and technical activities and S-14 Health services, whilst the rest of industrial sectors slightly benefit from applying the mix of benefits.

## Conclusions

Our results show that 7,205.43 million euros of LTC spending generates a total output of 10,266.88 million euros, maintaining 151,353 jobs, most of which belong to S15—Social work activities (65.08%), and which have been generated mainly as direct jobs from in-kind services (90.41%). The output generated entails 5,900 million euros in value added, mainly via the wages of sector S15—Social work activities (1,747 million euros) that are sustained by benefits granted as in-kind services. Moreover, the employment and value added generated would rise to 14,688 jobs and 8,603 million euros respectively if in-kind services were exclusively applied instead of the existing benefit-mix of CBPA, CBIC and in-kind services, according to the simulation here proposed.

To the best of our knowledge, this is the first study using an IO approach to assess the economy-wide impact of LTC spending on output, employment and value added. Furthermore, extending our IO model according to demographic-economic IO modelling enabled us to provide more accurate results since the effects of consumption of dependents, employed and unemployed were taken into consideration (Batey, 2018). The model herein applied additionally describes how the valued added generated by LTC expenditure is distributed according to the existing benefit-mix (in kind services, CBIC and CBPA). Lastly, as the LTC was originally designed to provide preferably in-kind services, a simulation exercise was performed to evaluate how to improve the return on LTC spending by using only in-kind services instead of the benefit-mix actually used.

IO methodology had previously been applied to estimate only the number of jobs required for the services provided by the LTC system (Herce et al., 2006). The authors of that study used the IO Table of 2000 from the National Institute of Statistics of Spain, concluding that by 2010, with the LTC system fully implemented, the LTC spending generated would be 33% higher than our estimation, resulting in a level of employment 30% higher. The results of our model provide more accurate information on the actual structure of LTC expenditure in Spain. By estimating the number of jobs sustained by LTC spending in different economic sectors, as well as the resulting level of output, income and taxes generated, our analysis provides a broader understanding of the significant role these transfers have in the Spanish economy, especially during the difficult times following the financial crisis. In addition, the outcomes from the simulation undertaken reveal that a full implementation of in-kind service would increase the total number of jobs by 77% compared to the benefit-mix actually carried out. In 2012, the unemployment rate in Spain rose by 25% (reaching a maximum of 27% in 2013) and GDP grew 0.42% (the sole year of positive growth between 2008 and 2013) (Quaglia & Royo, 2015). Our data support the need for a change in the structure of the services offered for LTCs to improve the efficiency of the system. These changes would involve an increase in in-kind services at the expense of cash benefits (mainly CBIC), which would lead to a greater generation of employment and fiscal returns for the Administration, consequently helping to alleviate the initial LTC spending.

Public expenditure on LTC is expected to grow from 0.8% in 2010 to 1.4% in 2060, while the leading countries in LTC investment, such as Sweden, Norway and the Netherlands, are already allocating up to 3% of its GDP (OECD, 2016). It can be ideal to allocate a percentage of public spending to LTC expenditure as it helps those who require assistance to carry out their day-to-day activities as well generating employment without any carry-over effects on other sectors. It also distributes more added value in the wages of workers and social contribution than the gross operating surplus (in-kind services versus cash benefits). However, it is worth mentioning that, considering our estimates, approximately 50% of the LTC expenditure is assumed by the dependent persons’ households through the co-payment of services. Reducing them as much as possible would be a good way to place Spain at the level of the most advanced countries in terms of LTC. These copayments (also referred to as out-of-pocket payments in the specific literature) can cause serious financial drawbacks in households (Wagstaff & van Doorslaer, 2003; Xu et al., 2003). In fact, a recent systematic review revealed that when co-payments are introduced, protection against catastrophic health care payments is needed for the most vulnerable groups (Kolasa and Kowalczyk 2016), thus evidencing why reducing co-payments must be encouraged. Among other ways, the incorporation and development of a series of financial tools from a public or private perspective or a public–private combination, such as the reverse mortgage, individual saving plans or the LTC insurance (Martinez-Lacoba et al., 2020) should be taken into consideration.

Some limitations need to be mentioned. Regarding the model and methodology used here, it should be noted that, while the main advantage of the model here presented is its ability to evaluate spill over effects in the local economy by measuring the linkages between industries in general equilibrium, the basic assumptions of IO methodology assume a fixed structure for each sector of the economy where no supply constraints are considered, industries present fixed output ratios and, therefore, economies of scale in production are ignored (Miller & Blair, 2009). It is also necessary to mention that, in the inputs of our model, only public expenses are included in the provision and not in the structure of the LTC system, such as the personnel involved in the administration and assessment of dependency.

Finally, it is worth noting that public spending modifies the macroeconomic growth potential of a country, in addition to helping to mitigate unfavorable economic situations or inequities in the distribution of wealth (Monsalve et al., 2019; Schwartz & Ter-Minassian, 2000). Ensuring that a country has economic structures, institutions, companies and people that are as resilient as possible to economic shocks contributes significantly to social well-being. The 2008 financial crisis showed the weakness of many countries (Duffie, 2017; Perez & Matsaganis, 2018) and the current crisis initiated by COVID-19 is acting as a stress test. In this sense, the countries of the EU are receiving economic support to mitigate the effects of the incipient crisis (Arbolino & Caro, 2021). Although this public spending is not necessarily a problem in itself, it is important to pay special attention to how it is implemented, possibly being an opportunity to improve the resilience of the countries' economies.

Our work shows that although public spending on LTC cannot be properly regarded as an investment, it allows people's well-being to be improved by sustaining employment and generating valued added that returns income to the economic institutions. We also show how it influences all industrial sectors through the spillover effect and we propose possible improvements in its efficiency based on the simulation carried out. Evidently, the economy of a country must focus on sectors with greater added value, but governments must also know how to meet people’s needs in order to achieve a more developed and equitable society through the optimization of public spending and in-depth knowledge of the operation of a system such as the LTC system.

## Supplementary Information

Below is the link to the electronic supplementary material.

Supplementary file1 (DOCX 86 kb)

## Data Availability

The data that support the findings of this study are available from the corresponding author upon reasonable request. The Stata syntax is available from the corresponding author upon reasonable request.

## Abbreviations

CBIC: Cash benefit for informal care
CBPA: Cash benefit for personal assistance
IO: Input–output
LTC: Long-term care

## Footnotes

1 For the sake of simplicity, the results of this study are shown at a disaggregated level in 16 sectors. Estimations for an additional 64 sectors disaggregation are available upon request.

2 Note that the recognition and management of the different LTC benefits are in hands of the regional governments, while other competences are assumed by the central government. Since the purpose of this study is to quantify the return of the expenditure allocated to LTC, the source of these public funds is not relevant in terms of the results. Consequently, we use the generic term of Administration throughout the text.
