# Supplementary material for: Impact of implementation of the Dependency Act on the Spanish economy: an analysis after the 2008 financial crisis
Source: Int J Health Econ Manag. 2021 Aug 4;22(1):111–28. doi: 10.1007/s10754-021-09310-9 (PMC8336904; doi:10.1007/s10754-021-09310-9)
Supplement: Supplementary file 1 — Supplementary file1 (DOCX 86 kb) [file 10754_2021_9310_MOESM1_ESM.docx]

**SUPPLEMENTARY MATERIAL**

**Figure S1. Multiplier effect of LTC spending on production, value added and employment according to the different types of benefit**

**Cash benefit for informal care**

**(CBIC)**

**In-kind services**

**Cash benefit for personal assistance**

**(CBPA)**

**x^D^ = 2.34**

**c^D^ = 1**

**l^D^ _tot_ = 43.83**

**l^D^ _dir_ = 30.18**

**l^D^ _indir_ = 3.82**

**l^D^ _induced_= 9.83**

**c^D^ = 1**

**c^D^ = 1**

**x^D^ = 1.78**

**x^D^ = 2.30**

**v^D^ = 0.99**

**w = 0.33**

**sc = 0.08**

**GOS = 0.55**

**NToP = 0.02**

**l^D^ _tot_ = 16.88**

**l^D^ _dir_ = 8.95**

**l^D^ _indir_ = 4.14**

**l^D^ _induced_= 3.79**

**v^D^ = 1.35**

**w = 0.83**

**sc = 0.23**

**GOS = 0.30**

**NToP = -0.02**

**l^D^ _tot_ = 41.91**

**l^D^ _dir_ = 28.67**

**l^D^ _indir_ = 3.84**

**l^D^ _induced_= 9.40**

**v^D^ = 1.37**

**w = 0.87**

**sc = 0.25**

**GOS = 0.29**

**NToP = -0.02**

**c^D^**: consumption funded by LTC spending

**x^D^**, **v^D^**, **l^D^**: production, value added and employment generated by consumption **c^D^**

**w**: wages;  **sc**: social contribution; **GOS**: Gross operating surplus; **NToP**: Net taxes on production;

**Table S1. Correspondence table from TSIO to 16 sectors in the model**

| **TSIO (ISIC rev4)** | **R-16** | |
| --- | --- | --- |
| Crop and animal production, hunting and related service activities | **S1** | Agriculture, forestry and fishing |
| Forestry and logging |  |  |
| Fishing and aquaculture |  |  |
| Mining and quarrying | **S2** | Energy supply, water supply and waste management activities |
| Electricity, gas, steam and air conditioning supply |  |  |
| Water collection, treatment and supply |  |  |
| Sewerage; waste collection, treatment and disposal activities; materials recovery; remediation activities and other waste management services |  |  |
| Manufacture of food products, beverages and tobacco products | **S3** | Food, beverages, tobacco and textiles |
| Manufacture of textiles, wearing apparel and leather products |  |  |
| Manufacture of wood and of products of wood and cork, except furniture; manufacture of articles of straw and plaiting materials | **S4** | Manufacture |
| Manufacture of paper and paper products |  |  |
| Printing and reproduction of recorded media |  |  |
| Manufacture of coke and refined petroleum products |  |  |
| Manufacture of chemicals and chemical products |  |  |
| Manufacture of basic pharmaceutical products and pharmaceutical preparations |  |  |
| Manufacture of rubber and plastic products |  |  |
| Manufacture of other non-metallic mineral products |  |  |
| Manufacture of basic metals |  |  |
| Manufacture of fabricated metal products, except machinery and equipment |  |  |
| Manufacture of computer, electronic and optical products |  |  |
| Manufacture of electrical equipment |  |  |
| Manufacture of machinery and equipment n.e.c. |  |  |
| Manufacture of motor vehicles, trailers and semi-trailers |  |  |
| Manufacture of other transport equipment |  |  |
| Manufacture of furniture; other manufacturing |  |  |
| Repair and installation of machinery and equipment |  |  |
| Construction | **S5** | Construction |
| Wholesale and retail trade and repair of motor vehicles and motorcycles | **S6** | Wholesale and retail trade |
| Wholesale trade, except of motor vehicles and motorcycles |  |  |
| Retail trade, except of motor vehicles and motorcycles |  |  |
| Land transport and transport via pipelines | **S7** | Transport |
| Water transport |  |  |
| Air transport |  |  |
| Warehousing and support activities for transportation |  |  |
| Postal and courier activities |  |  |

| **TSIO (ISIC rev4)** | **R-16** | |
| --- | --- | --- |
| Accommodation and food service activities | **S8** | Accommodation and food service activities |
| Publishing activities | **S9** | Information and communication |
| Motion picture, video and television programme production, sound recording and music publishing activities; programming and broadcasting activities |  |  |
| Telecommunications |  |  |
| Computer programming, consultancy and related activities; information service activities |  |  |
| Financial service activities, except insurance and pension funding | **S10** | Financial and insurance activities |
| Insurance, reinsurance and pension funding, except compulsory social security |  |  |
| Activities auxiliary to financial services and insurance activities |  |  |
| Real estate activities | **S11** | Real estate activities |
| Legal and accounting activities; activities of head offices; management consultancy activities | **S12** | Professional, scientific and technical activities; administrative and support service activities |
| Architectural and engineering activities; technical testing and analysis |  |  |
| Scientific research and development |  |  |
| Advertising and market research |  |  |
| Other professional, scientific and technical activities; veterinary activities |  |  |
| Administrative and support service activities |  |  |
| Public administration and defence; compulsory social security | **S13** | Public administration, defence and education |
| Education |  |  |
| Human health and social work activities | **S14** | Health services |
|  | **S15** | Social work activities |
| Other service activities | **S16** | Arts, entertainment and recreation; other service activities; activities of household and extra-territorial organizations and bodies |
| Activities of households as employers; |  |  |
| Activities of extraterritorial organizations and bodies |  |  |

**Table S2. Correspondence table from 16 sectors in the model to COICOP in HBS**

| **R-16** | **COICOP in HBS** | |
| --- | --- | --- |
| **S01** | 0116x | Fruit |
|  | 0117x | Vegetables |
|  | 0933x | Gardens, plants and flowers |
| **S02** | 0441x | Water supply |
|  | 0442x | Refuse collection |
|  | 0443x | Sewerage collection |
|  | 0451x | Electricity |
|  | 0452x | Gas |
|  | 0454x | Solid fuels |
|  | 0455x | Heat energy |
| **S03** | 0111x | Bread and cereals |
|  | 0112x | Meet |
|  | 0113x | Fish and seafood |
|  | 0114x | Milk, cheese and eggs |
|  | 0115x | Oils and fats |
|  | 0118x | Sugar, jam, honey, chocolate and confectionery |
|  | 0119x | Food products n.e.c. |
|  | 012xx | Non-alcoholic beverages |
|  | 021xx | Alcoholic beverages |
|  | 022xx | Tobacco |
|  | 0311x | Clothing materials |
|  | 0312x | Garments |
|  | 0313x | Other articles of clothing and clothing accessories |
|  | 0321x | Shoes and other footwear |
|  | 0512x | Carpets and other floor coverings |
|  | 052xx | Household textiles |
| **S04** | 0231x | Narcotics |
|  | 0431x | Materials for the maintenance and repair of the dwelling |
|  | 0453x | Liquid fuels |
|  | 0511x | Furniture and furnishings |
|  | 0531x | Major household appliances whether electric or not |
|  | 0532x | Small electric household appliances |
|  | 054xx | Glassware, tableware and household utensils |
|  | 055xx | Tools and equipment for house and garden |
|  | 0561x | Non-durable household goods |
|  | 061xx | Medical products, appliances and equipment |
|  | 071xx | Purchases of vehicles |
|  | 0721x | Spare parts and accessories for personal transport equipment |
|  | 0722x | Fuels and lubricants for personal transport equipment |
|  | 082xx | Telecommunication equipment |
|  | 091xx | Audio-visual, photographic and information processing equipment |
|  | 092xx | Other major durables for recreation and culture |
|  | 0931x | Games, toys and hobbies |
|  | 0932x | Equipment for sport, camping and open-air recreation |
|  | 095xx | Newspapers, books and stationery |
|  | 1212x | Electric appliances for personal care |
|  | 1213x | Other appliances, articles and products for personal care |
|  | 122xx | Jewellery, clocks, watches and other personal effects |
| **S05** | 0432x | Services for the maintenance and repair of the dwelling |

| **R-16** | **COICOP in HBS** | |
| --- | --- | --- |
| **S06** | 0723x | Maintenance and repair of personal transport equipment |
|  | 0724x | Other services in respect of personal transport equipment |
| **S07** | 073xx | Transport services |
|  | 081xx | Postal services |
| **S08** | 111xx | Catering services |
|  | 112xx | Accommodation services |
| **S09** | 083xx | Telecommunication services |
| **S10** | 124xx | Insurance |
|  | 125xx | Financial services |
| **S11** | 041xx | Actual rentals paid by tenants |
|  | 042xx | Imputed rentals of owner-occupiers |
| **S12** | 0444x | Other services relating to the dwelling n.e.c. |
|  | 126xx | Other services n.e.c. |
| **S13** | 05622 | Compulsory social security |
|  | 10xxx | Education |
| **S14** | 062xx | Out-patient services |
|  | 063xx | Hospital services |
| **S15** | 123xx | Social protection |
| **S16** | 0314x | Cleaning, repair and hire of clothing |
|  | 0322x | Repair and hire of footwear |
|  | 0513x | Repair of furniture, furnishings and floor coverings |
|  | 0533x | Repair of household appliances |
|  | 05621 | Domestic services and household services |
|  | 094xx | Recreational and cultural services |
|  | 1211x | Hairdressing salons and personal grooming establishments |
|  | 1271x | Out-of-pocket money |
|  | 1281x |  |

**Table S3. Dependency contributions by Administration and beneficiaries. Total annual contributions by type of benefit (million euros 2012).**

|  | CASH BENEFITS | | IN-KIND SERVICES | TOTAL |
| --- | --- | --- | --- | --- |
|  | Cash Benefit for Personal Assistance (CBPA) | Cash Benefit for Informal Care (CBIC) |  |  |
| Contribution  by the Administration | 181.17 | 1,174.92 | 2,493.64 | 3,849.74 |
| Contribution  by beneficiary | 174.28 | 1,130.20 | 2,051.21 | 3,355.69 |
| Total contribution | 355.45 | 2,305.12 | 4,544.85 | 7,205.43 |

**Table S4. Consumption profile of dependents** **(**$\mathbf{c}_{D}$**)**, **employed** **(**$\mathbf{c}_{E}$**)** **and unemployed** **(**$\mathbf{c}_{U}$**)** **(2012)**

|  |  |  |  | | Cash benefits | | 2,660.57 | | In-kind services | 4,544.85 |
| --- | --- | --- | --- | --- | --- | --- | --- | --- | --- | --- |
|  |  |  |  | CBPA | | 355.45 | CBIC | 2,305.12 |  |  |
|  |  |  |  | | Administration | 181.17 | Administration | 1,174.92 | Administration | 2,493.64 |
|  |  |  |  | | Households | 174.28 | Households | 1,130.20 | Households | 2,051.21 |
| Sector | $\mathbf{c}_{D}$ (%) | $\mathbf{c}_{E}$ (%) | $\mathbf{c}_{U}$ (%) | | $\mathbf{c}^{\mathbf{D}}$ (million euros) | | $\mathbf{c}^{\mathbf{D}}$ (million euros) | | $\mathbf{c}^{\mathbf{D}}$ (million euros) | |
| S1 | 1.11% | 0.90% | 1.09% | | -1.27 | | 16.80 | | -14.95 | |
| S2 | 4.18% | 3.60% | 4.51% | | -6.95 | | 91.88 | | -81.76 | |
| S3 | 5.68% | 5.05% | 5.45% | | -6.06 | | 80.11 | | -71.29 | |
| S4 | 4.63% | 5.17% | 4.36% | | -4.78 | | 63.27 | | -56.30 | |
| S5 | 1.24% | 1.13% | 0.88% | | -2.07 | | 27.33 | | -24.32 | |
| S6 | 20.10% | 21.11% | 22.33% | | -32.32 | | 427.46 | | -380.38 | |
| S7 | 2.33% | 3.31% | 2.15% | | -3.59 | | 47.45 | | -42.22 | |
| S8 | 13.78% | 19.45% | 12.18% | | -22.99 | | 304.08 | | -270.59 | |
| S9 | 3.14% | 3.19% | 3.50% | | -4.94 | | 65.35 | | -58.15 | |
| S10 | 3.16% | 3.50% | 3.02% | | -4.97 | | 65.75 | | -58.51 | |
| S11 | 25.26% | 18.66% | 30.84% | | -42.30 | | 559.51 | | -497.88 | |
| S12 | 1.52% | 1.35% | 1.15% | | -2.10 | | 27.72 | | -24.67 | |
| S13 | 2.37% | 3.65% | 1.53% | | -3.94 | | 52.12 | | -46.38 | |
| S14 | 3.16% | 2.95% | 2.68% | | -5.28 | | 69.82 | | -62.13 | |
| S15 | 1.99% | 0.85% | 0.53% | | 343.35 | | 43.95 | | 4,393.44 | |
| S16 | 6.34% | 6.12% | 3.80% | | -10.18 | | 134.64 | | -119.81 | |
| **Total** | **100.00%** | **100.00%** | **100.00%** | | **189.62** | | **2,077.26** | | **2,584.10** | |

*Source*: Authors’ own calculations from HBS data [51].

Note: S1. Agriculture, forestry and fishing; S2. Energy, water and waste collection; S3. Food and textile; S4. Manufacturing; S5. Construction; S6. Wholesale and retail trade; S7. Transport; S8. Accommodation and food service activities; S9. Information and communication; S10. Financial and insurance activities; S11. Real estate activities; S12. Professional, scientific and technical activities; S13. Public administration, defence and education; S14. Health services; S15. Social work activities; S16. Arts, entertainment, recreation and other services.

$\mathbf{c}_{D}$, $\mathbf{c}_{E}$, $\mathbf{c}_{U}$: Distribution of consumption by dependents, employed and unemployed according to the HBS

CBPA: Cash benefit for personal assistance; CBIC: Cash benefit for informal care.

$\mathbf{c}^{\mathbf{D}}$: Monetary values of consumption in producer prices according to the different types of LTC benefits.

**Table S5. Demo-economic return considering in-kind services had been exclusively granted (2012)**

| Sector | Total Output  $\mathbf{x}^{D}$ | Total  Value added  $\mathbf{v}^{D}$ | Wages | Social  Contrib | GOS | NToP | $\mathbf{l}^{D}$ | $\mathbf{l}^{D}$_dir_ | $\mathbf{l}^{D}$_indir_ | $\mathbf{l}^{D}$_indu_ |
| --- | --- | --- | --- | --- | --- | --- | --- | --- | --- | --- |
| S1 | 59.88 | 31.51 | 5.01 | 0.64 | 32.96 | -7.10 | 968 | -624 | -370 | 1,961 |
| S2 | 225.98 | 77.73 | 17.55 | 4.35 | 54.48 | 1.34 | 475 | -443 | 214 | 704 |
| S3 | 317.79 | 69.36 | 27.58 | 6.41 | 35.41 | -0.04 | 1,212 | -702 | -3 | 1,917 |
| S4 | 953.00 | 232.21 | 111.52 | 28.84 | 92.42 | -0.56 | 3,709 | -566 | 2,190 | 2,085 |
| S5 | 107.38 | 47.42 | 18.63 | 4.98 | 22.87 | 0.94 | 858 | -502 | 365 | 994 |
| S6 | 392.23 | 228.24 | 108.12 | 28.94 | 90.63 | 0.54 | 6,654 | -16,656 | 7,163 | 16,148 |
| S7 | 221.40 | 89.42 | 39.55 | 10.70 | 39.13 | 0.04 | 1,749 | -861 | -208 | 2,818 |
| S8 | 264.95 | 152.99 | 61.45 | 9.31 | 81.68 | 0.55 | 3,087 | -8,137 | 1,186 | 10,037 |
| S9 | 175.68 | 85.25 | 33.58 | 8.57 | 42.30 | 0.80 | 933 | -797 | 491 | 1,239 |
| S10 | 144.32 | 83.20 | 33.03 | 11.28 | 31.86 | 7.03 | 795 | -832 | -42 | 1,669 |
| S11 | -683.58 | -586.89 | -18.00 | -4.61 | -521.14 | -43.14 | -977 | -1,837 | -58 | 917 |
| S12 | 449.52 | 255.00 | 136.54 | 37.59 | 81.89 | -1.02 | 7,572 | -1,073 | 3,658 | 4,987 |
| S13 | 135.26 | 105.74 | 61.96 | 19.06 | 24.56 | 0.16 | 2,387 | -2,112 | 699 | 3,800 |
| S14 | 428.31 | 273.67 | 168.63 | 42.67 | 62.02 | 0.34 | 5,143 | -1,926 | 5,497 | 1,573 |
| S15 | 11,446.99 | 7,426.28 | 4,486.76 | 1,284.22 | 1,751.36 | -96.06 | 232,030 | 228,661 | 2,533 | 836 |
| S16 | 48.91 | 31.55 | 17.73 | 3.54 | 10.26 | 0.01 | 1,305 | -8,253 | 1,159 | 8,399 |
| **Total** | **14,688.01** | **8,602.66** | **5,309.64**  **(61.7%)** | **1,496.48**  **(17.4%)** | **1,932.70**  **(22.5%)** | **-136.16**  **(-1.6%)** | **267,899** | **183,341**  **(68.4%)** | **24,472**  **(9.1%)** | **60,086**  **(22.4%)** |

Note: Monetary values are denoted in million euros

S1. Agriculture, forestry and fishing; S2. Energy, water and waste collection; S3. Food and textile; S4. Manufacturing; S5. Construction; S6. Wholesale and retail trade; S7. Transport; S8. Accommodation and food service activities; S9. Information and communication; S10. Financial and insurance activities; S11. Real estate activities; S12. Professional, scientific and technical activities; S13. Public administration, defence and education; S14. Health services; S15: Social work activities; S16. Arts, entertainment, recreation and other services

$\mathbf{x}^{\mathbf{D}}$, $\mathbf{v}^{\mathbf{D}}$: Million euros of production and value added

${\mathbf{l}^{D}}_{\mathbf{dir}}$: Direct employment; ${\mathbf{l}^{D}}_{\mathbf{indir}}$: Indirect employment; ${\mathbf{l}^{D}}_{\mathbf{indu}}$: Induced employment; $\mathbf{l}^{D}$: Total employment
